# Benchmarking computational variant effect predictors by their ability to infer human traits

**DOI:** 10.1186/s13059-024-03314-7

**Published:** 2024-07-01

**Authors:** Daniel R. Tabet, Da Kuang, Megan C. Lancaster, Roujia Li, Karen Liu, Jochen Weile, Atina G. Coté, Yingzhou Wu, Robert A. Hegele, Dan M. Roden, Frederick P. Roth

**Affiliations:** 1 Donnelly Centre, University of Toronto, Toronto, ON Canada; 2 Department of Molecular Genetics, University of Toronto, Toronto, ON Canada; 3 Department of Computer Science, University of Toronto, Toronto, ON Canada; 4 Lunenfeld-Tanenbaum Research Institute, Sinai Health, Toronto, ON Canada; 5 Division of Cardiovascular Medicine, Department of Medicine, Vanderbilt University Medical Center, Nashville, TN USA; 6 Department of Pharmacology, Vanderbilt University Medical Centre, Nashville, TN USA; 7 Department of Biomedical Informatics, Vanderbilt University Medical Center, Nashville, TN USA; 8 Department of Medicine, Department of Biochemistry, Schulich School of Medicine and Dentistry, Robarts Research Institute, Western University, London, ON Canada; 9 Department of Computational and Systems Biology, University of Pittsburgh School of Medicine, Pittsburgh, PA USA

**Keywords:** Variant effect predictors, Rare missense variation, Benchmarking, Personal genomics, UK Biobank, All of Us

## Abstract

**Background:**

Computational variant effect predictors offer a scalable and increasingly reliable means of interpreting human genetic variation, but concerns of circularity and bias have limited previous methods for evaluating and comparing predictors. Population-level cohorts of genotyped and phenotyped participants that have not been used in predictor training can facilitate an unbiased benchmarking of available methods. Using a curated set of human gene-trait associations with a reported rare-variant burden association, we evaluate the correlations of 24 computational variant effect predictors with associated human traits in the UK Biobank and *All of Us* cohorts.

**Results:**

AlphaMissense outperformed all other predictors in inferring human traits based on rare missense variants in UK Biobank and *All of Us* participants. The overall rankings of computational variant effect predictors in these two cohorts showed a significant positive correlation.

**Conclusion:**

We describe a method to assess computational variant effect predictors that sidesteps the limitations of previous evaluations. This approach is generalizable to future predictors and could continue to inform predictor choice for personal and clinical genetics.

**Supplementary Information:**

The online version contains supplementary material available at 10.1186/s13059-024-03314-7.

## Background

The increasing accessibility of genetic sequencing has ushered in a new era of personal and clinical genomics. However, a central challenge remains: the phenotypic impact of genetic variation at the organismal level cannot be reliably inferred from sequence. Given both the pace and promise of human genome sequencing, the need for scalable evidence to aid in variant interpretation is critical. While experimental functional assessments can provide evidence of variant effects [51], these measurements remain sparse, with a comprehensive atlas of experimental data being far from complete. As a ready alternative, computational variant effect predictors (hereafter referred to as predictors) [24, 31] offer an increasingly reliable and already nearly comprehensive means of interpreting human genetic variation—though it remains uncertain which predictors are best suited to this task.

Although many efforts to compare predictor performance have been reported, confidence in their results has been persistently limited by concerns of circularity and bias. More specifically, where training data is skewed towards pathogenic or benign variants (e.g., within proteins or protein families) or where training data is later re-used (either directly or indirectly) in evaluation, performance estimates for a given predictor may be artificially inflated depending on the benchmark set of choice [16, 31]. Given the breadth of training data used by the many available predictors, few objective ground truth sets remain.

In an initial effort to limit concerns of circularity, Livesey and Marsh benchmarked predictor performance against large-scale sequence-function data in the form of ‘variant effect maps’ [30]. Unfortunately, such maps are available for less than 1% of human genes [11] and, in some instances, have already been used in model training (e.g., DeepSequence and VARITY) [30, 42, 58]. More generally, benchmarking strategies have been primarily based on either clinically classified variants, which (in addition to having been used in training sets) may in themselves be biased, or on biophysical evidence, which, importantly, may not correspond to human phenotypic endpoints.

To facilitate a broader and unbiased benchmarking of computational variant effect predictors, we describe the use of population-level cohorts of genotyped and phenotyped participants (that have not been used in predictor training). We evaluate the performance of 24 computational variant effect predictors against a set of previously reported gene-trait associations using exome-sequenced UK Biobank participants (UK Biobank) [4] and confirm our findings using an independent whole-genome sequenced human cohort from *All of Us* [2].

## Results

### Evaluating the performance of computational variant effect predictors in the UK Biobank

**Fig. 1:**
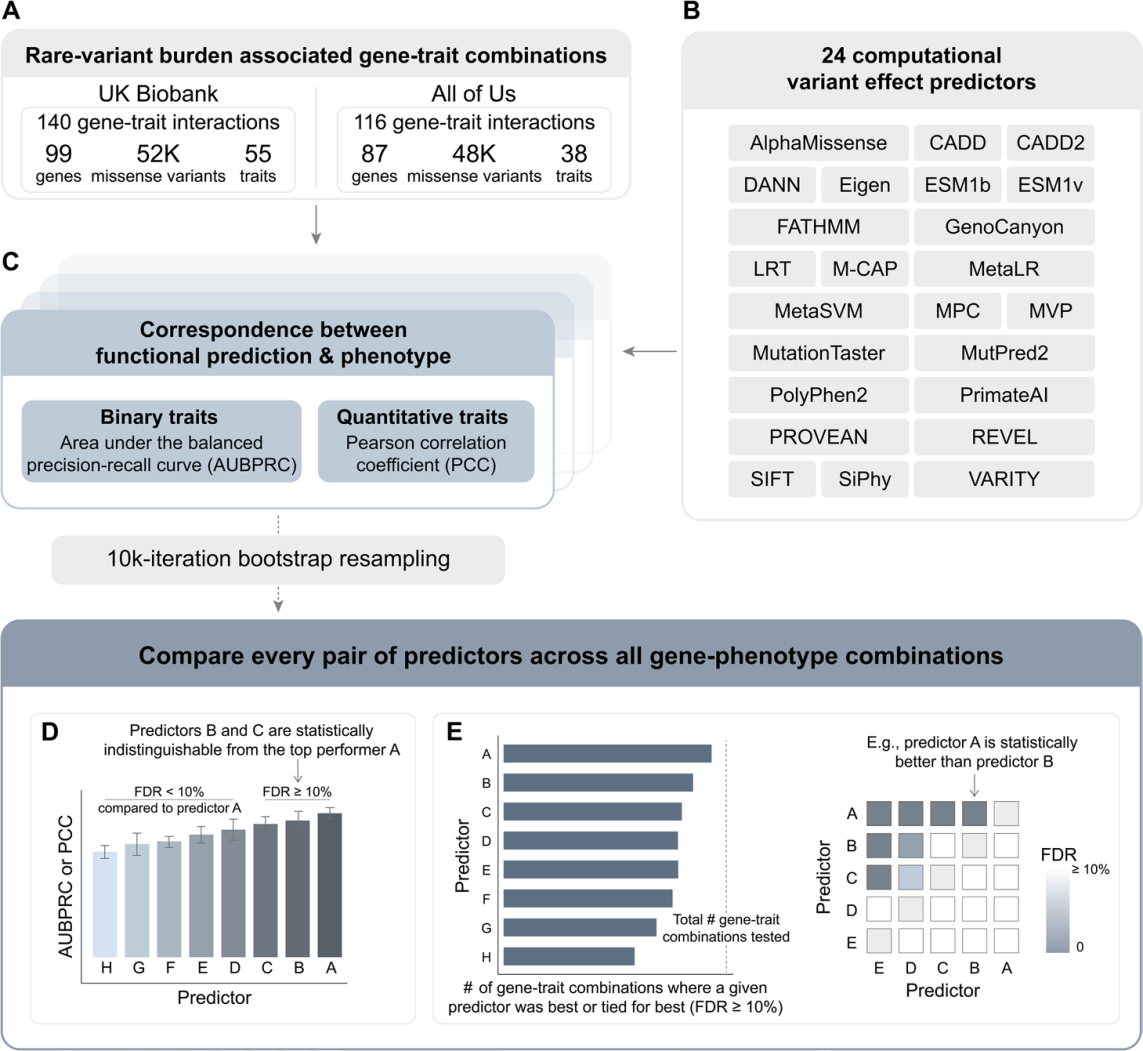
Schematic overview of predictor benchmarking in population-based cohorts based on human gene-trait associations. **A** Participant-level genotypes and phenotypes were extracted from the UK Biobank and *All of Us* cohorts for the corresponding sets of gene-trait combinations, and (**B**) predicted functional scores were collected for a set of 24 computational variant effect predictors. **C** In order to assess predictor performance, the area under the balanced precision-recall curve (AUBPRC) and Pearson correlation coefficient (PCC) was measured for binary and quantitative traits, respectively. To estimate the uncertainty in these measurements, participants were resampled with replacement and performance measures recalculated for each resampled set. **D** For each gene-trait combination, predictors were ranked by mean performance (AUBPRC or PCC), and a false discovery rate (FDR) was calculated to assess whether performance differences were statistically significant. **E** To summarize comparisons across all gene-trait combinations, we (left) summed the number of combinations for which a predictor was either best performing or tied (FDR ≥ 10%) for best, and (right) compared the overall difference in performance measures between predictor pairs across all gene-trait combinations

To assess the correspondence of predicted functional scores with human phenotypes (Fig. 1), we 1) assembled 140 gene-trait combinations previously reported in rare-variant burden association studies in the UK Biobank (Additional file 1: Table S1) [8, 10, 22, 54], 2) extracted rare missense variants from the corresponding 99 genes; and 3) collected predicted functional scores from 24 computational variant effect predictors (Additional file 2: Table S2) [1, 3, 5–7, 12, 15, 19–21, 26, 33–35, 38, 39, 43–46, 49, 55, 58].

Of the 99 trait-associated genes, 73 were associated with only one trait and the remaining 26 were associated with multiple traits. For instance, *LDLR* [17], which encodes the low-density lipoprotein (LDL) receptor, was associated with 5 traits, each relating to circulating LDL levels, statin use, or atherosclerotic heart disease (UK Biobank field IDs 30,780, 20,002–1473, 20,003–1141146234, 6153–1 and 41270-I25).

We extracted missense variants for the 99 trait-associated genes from the UK Biobank whole-exome release (UK Biobank field ID 23157). Since clinical and experimental evidence is especially sparse for rare variants, we restricted our benchmarking effort to variants with a minor allele frequency (MAF) < 0.1%. In this range, predictor performance is especially critical [47] given that rare variants are more likely than common variants to have large phenotypic effects [9].

We obtained predicted functional scores from the 24 predictors, finding that each provided scores for more than 90% of the missense variants in the UK Biobank set (Additional file 3: Table S3). In evaluating performance for individual genes, we only considered predictors making 10 or more predictions for a given gene; for 84/99 genes, this criterion was met by all 24 predictors (Additional file 4: Table S5). We had initially intended to evaluate EVE [14] but because it only provided predictions for 41% of the variants in our set and provided no scores at all for more than half of the 99 genes, it was not included.

We measured predictor performance using one of two methods, depending on whether a trait was binary or quantitative. For gene-trait combinations for which the trait was binary [including categorical traits that could be treated as binary (e.g., self-reported medication use; UK Biobank field ID 6153)], we evaluated the area under the balanced precision-recall curve (AUBPRC). Here, precision is the fraction of positive predictions (i.e., predictions that a given participant has a given trait) that are correct, and recall is the fraction of participants with the given trait that were detected. More specifically, because the precision measure is affected by the prevalence of positive events, we evaluated the balanced precision—defined as the precision when the prior probability of a positive event is 50% [58]. Where participants carried multiple missense variants in a given gene, we took the sum of predicted scores, an approach that models variant effects as though they are additive. We note that, because only ~ 1% of participants carried multiple variants in a given gene, more sophisticated models would be unlikely to alter our results. For quantitative traits, we assessed the correspondence between predicted variant impact and trait value using the Pearson Correlation Coefficient (PCC). Where multiple participants carried the same variant for a given trait, quantitative values were averaged.

To estimate the uncertainty in each of these performance measures, we carried out a 10 k-iteration bootstrap resampling, in which participants were re-sampled with replacement and performance measures recalculated for each resampled set. For each gene-trait combination and every predictor, this yielded a distribution of performance measures from which we extracted the mean and 95% confidence interval (CI).

**Fig. 2:**
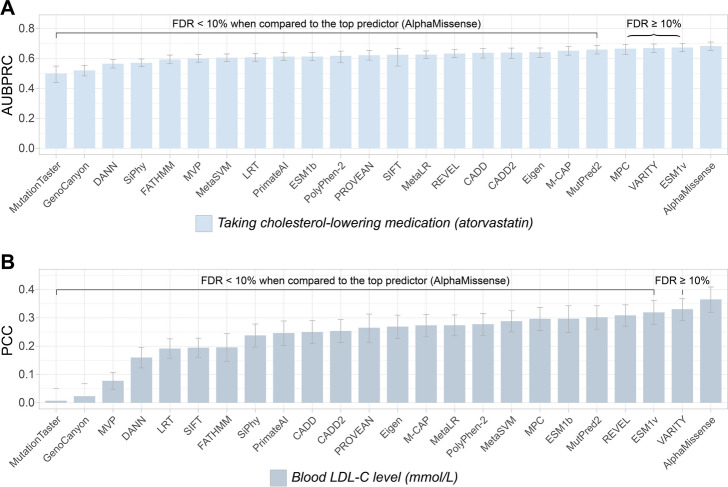
The performance of 24 computational variant effect predictors in predicting two cholesterol-related phenotypes based on the presence of rare LDLR missense variants*.* Performance comparisons measured the ability of predictors to infer **(A)** whether participants were taking the cholesterol-lowering medication atorvastatin (AUBPRC) and **(B)** circulating LDL-C levels (PCC) based on participant *LDLR* genotype. Mean performance measures were derived from a 10 k-iteration bootstrap resampling, error bars indicate the 95th percentile confidence interval. In each ranking, predictors that were statistically indistinguishable (FDR ≥ 10%) from the top predictor (AlphaMissense) are indicated

To assess whether performance differences between predictors were statistically significant, we calculated a *p*-value for every pairwise combination of predictors for each of the 140 gene-trait combinations. Here, for each predictor pair (e.g., predictors *a* and *b*), our empirical *p*-value is the fraction of resampled participant sets in which predictor *a* is outperformed by predictor *b*. This generated a distribution of *p*-values, one for every pairwise comparison, from which we calculated Storey’s *q*-values to measure the false discovery rate (FDR) [48]. We consider a given predictor to significantly outperform another if the comparison yielded an FDR (i.e., a *q*-value) < 10%. To illustrate this approach, we show the AUBPRC and PCC values for all 24 predictors for a binary phenotype (use of the cholesterol-lowering medication atorvastatin) and a quantitative phenotype (blood LDL-C level) associated with *LDLR* (Fig. 2). For both gene-trait combinations, AlphaMissense was the top-performing predictor; however, it was statistically indistinguishable (FDR ≥ 10%) from ESM-1v, VARITY, and MPC in inferring atorvastatin use, and indistinguishable from VARITY in inferring LDL-C.

To summarize similar comparisons across all 140 gene-trait combinations, we summed the number of combinations for which a predictor was either best performing (according to mean AUBPRC or PCC) or was tied (i.e., yielded an FDR ≥ 10% in the comparison) with the numerically best-performing predictor. To assess the significance of the overall difference in performance measures between predictor pairs, we performed a two-tailed Wilcoxon signed-rank test comparing the distributions of mean performance measures across all gene-trait combinations for each pair of predictors. From this test statistic, we extracted *p*-values and again calculated Storey’s *q*-values to estimate the false discovery rate, judging performance to be statistically different at an FDR < 10%. Where predictors were tied (i.e., were best or tied for best in the same number of gene-trait combinations), ties were broken first based on the number of pairwise comparisons for which a given predictor statistically outperformed another across all gene-trait combinations; and second, where necessary, based on the number of comparisons for which a given predictor yielded lower *q*-values than the predictor with which it was tied. Thus, we assessed both the number of gene-trait combinations in which each predictor was either best or tied for best and also, for each pair of predictors, directly assessed differences in performance across all gene-trait combinations.

**Fig. 3:**
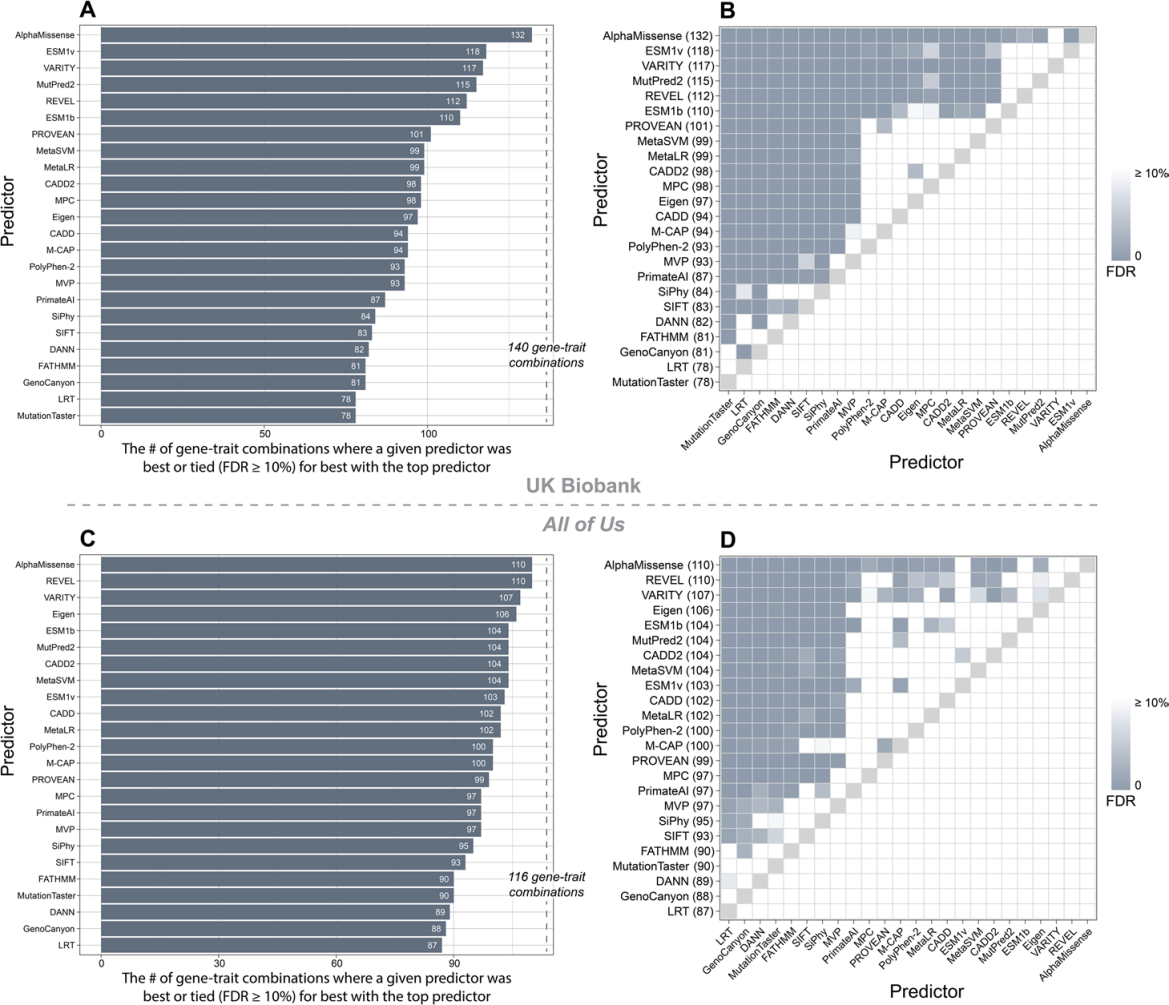
Predictor rankings across all gene-trait combinations in the UK Biobank (top) and *All of Us* (bottom) cohorts. **A** and** C** The number of gene-trait combinations for which a given predictor was either best performing (in terms of mean AUBPRC or PCC) or tied (FDR ≥ 10%) with the best-performing predictor in the UK Biobank and *All of Us* cohorts, respectively. In the UK Biobank cohort, 140 gene-trait combinations were considered; from this, 116 gene-trait combinations were matched in the *All of Us* cohort. **B** and **D** The overall difference in performance measures between predictor pairs was assessed using a two-tailed Wilcoxon signed-rank test comparing the distributions of mean performance measures across all gene-trait combinations for each pair; the predictors in a given pair are considered statistically different at an FDR < 10% (indicated in blue-grey). Where predictors were tied in the overall ranking (i.e., were best or tied for best in the same number of gene-trait combinations) ties were broken first based on the number of pairwise comparisons for which a given predictor statistically outperformed another across all gene-trait combinations; and second, where necessary, based on the number of comparisons for which a given predictor yielded lower *q*-values than the predictor with which it was tied. The overall ranking of predictors in the UK Biobank and *All of Us* cohorts showed significant positive correlation (Kendall’s Tau = 0.75; *p*-value = 1 × 10^–8^)

In benchmarking all 24 predictors for each of the 140 gene-trait combinations in the UK Biobank cohort, we found AlphaMissense to be either best (or tied for best) in 132 (out of 140) gene-trait combinations, exceeding all other predictors (Fig. 3A). Moreover, the pairwise comparison between AlphaMissense and each other predictor, which evaluated the difference in the distribution of performance measures across all gene-trait combinations, found that AlphaMissense yielded significantly higher performance than each other predictor, with the exception of VARITY, for which the FDR was > 10% (*q*-value = 0.16) (Fig. 3B).

### Evaluating the performance of computational variant effect predictors in *All of Us*

We next sought to benchmark predictors in the independent whole-genome sequenced and phenotyped *All of Us* cohort of 245,400 participants. Of the 140 gene-trait combinations in our UK Biobank analysis, 116 had matching phenotypes in *All of Us* (Additional file 1: Table S1). We extracted missense variants (MAF < 0.1%) for these genes and assembled functional scores from the same 24 predictors evaluated above. Each predictor provided scores for more than 90% of missense variants in the *All of Us* set (Additional file 3: Table S4*)*, and 74/87 genes had 10 or more predictions from all 24 predictors (Additional file 4: Table S5).

We measured predictor performance in *All of Us* for each gene-trait combination as above (i.e., AUBPRC or PCC) and recalculated performance measures for each of the 10 k resampled sets of participants. From this, we again determined the number of gene-trait combinations for which each predictor performed best or was statistically indistinguishable from the best predictor, and assessed the statistical significance of quantitative differences in performance across all gene-trait combinations as was done for the UK Biobank cohort.

In benchmarking all 24 predictors for each of the 116 gene-trait combinations in *All of Us*, we found AlphaMissense and REVEL to be either the best (or tied for best) in 110 (out of 116) gene-trait combinations, which was more than every other predictor (Fig. 3C). Ties were again broken, as described above. In comparing the quantitative performance of predictors across all gene-trait combinations, we found that AlphaMissense statistically outperformed all predictors except REVEL (*q*-value = 0.99), VARITY (*q*-value = 0.22), Esm1b (*q*-value = 0.41) and Esm-1v (*q*-value = 0.21) for which FDRs exceeded our cutoff of 10% (Fig. 3D). Although AlphaMissense and REVEL were tied (i.e., both were best or tied for best in 110/116 gene-trait combinations), AlphaMissense was statistically better than 19 of the 23 other predictors, whereas REVEL only statistically outperformed 16 out of the 23 other predictors. Thus, we again deem AlphaMissense to be the top-performing predictor. The overall ranking of computational variant effect predictors in the UK Biobank and *All of Us* cohorts showed significant positive correlation (Kendall’s Tau = 0.75; *p*-value = 1 × 10^–8^), with AlphaMissense being the top performer in both rankings. Overall rankings were also similar among lesser-performing predictors, with the bottom 7 predictors in the UK Biobank set all falling amongst the bottom 8 predictors in *All of Us*. Thus, the evaluation of predictors in the independent *All of Us* cohort yielded results consistent with those in UK Biobank.

## Discussion

Here, we evaluate the performance of 24 computational variant effect predictors using a set of rare-variant burden associated gene-trait combinations in the UK Biobank and *All of Us* cohorts. Because none of the computational predictors we assessed had been trained on data from either of these sources, our evaluation avoids the issues of performance inflation that can arise when a predictor is benchmarked against an evaluation set on which it has previously been trained (i.e., circularity). By assessing each predictor across a range of human genes and traits, we were able to determine which predictor performed best overall in a comprehensive ranking.

Notably, the top-performing predictor(s) in the UK Biobank and *All of Us* cohorts were recently-developed unsupervised predictors (i.e., AlphaMissense, ESM1b, and ESM-1v), which substantially outperformed previous unsupervised methods. Among the top predictors, several were meta-predictors (i.e., MutPred2, REVEL, and VARITY), which integrate the outputs of other predictors as features in their own predictions. It can be particularly challenging to establish ground truth sets for meta-predictors that have not been used in training either for the predictor itself or in training for any of the predictors used as features. That said, the predictors used as input for VARITY were limited to unsupervised methods (i.e., predictors that made no direct use of clinical annotations).

One application where computational variant effect predictors are particularly useful is in improving the detection of gene-trait associations in burden association studies, which seek differences in the observed frequency (i.e., burden) of genetic variation at particular genetic loci between people with (or without) a given trait [28, 36]. Predictors can improve the correlation between traits and variant burden by filtering out milder or neutral variants that are less likely to affect a given trait. By benchmarking predictors against population phenotypes, our study facilitates an informed selection of top predictors for this task, the use of which should improve both the power and accuracy of future burden association tests.

We acknowledge limitations of our study. First, we note that various predictors had previously been employed in the original rare-variant burden analyses that led us to select the 140 gene-trait combinations for the evaluation. Indeed, this might tend to advantage the predictors used in the original identification of the 140 gene-trait combinations and may well have affected the relative performance of some pairs of predictors. However, most of the top-performing predictors (i.e., AlphaMissense, ESM1b, ESM-1v, MutPred2, and VARITY) had not been used in any of the rare-variant burden scans on which we relied to choose the 140 gene-trait combinations.

Second, we did not evaluate all known published predictors, mainly due to the sheer number of available methods. We were, however, able to assess many widely used predictors and several recent predictors reported to have superior performance. Notably absent from our study is the predictor EVE, which was excluded because it provided too few scores to be assessed fairly.

Third, we did not consider the correlation between traits. For instance, gene-trait combinations for the gene *LDLR* included multiple interrelated traits, and so our analysis will have been influenced by some phenotypic endpoints more than others. That said, no one gene or trait disproportionately dominated the set of 140 gene-trait combinations: body mass index was the most recurrent trait (involved in 23 gene-trait combinations) and *LDLR* was the most recurrent gene (involved in 5 gene-trait combinations).

It may cause concern for some that many UK Biobank and *All of Us* participants carried variants that will have been seen in training by many of the predictors in our assessment. However, it is unlikely that many of these variants would have been deposited or annotated in ClinVar or the Human Gene Mutation Database (HGMD) on the basis of having been observed in either of these population cohorts, especially given that we excluded common variants. Taken together with the fact that the traits of UK Biobank and *All of Us* participants arose independently from knowledge of pre-existing variant annotations, the ability of a predictor to infer traits in these cohorts cannot be attributed to overfitting.

It is very likely that (with the addition of more nuance to our analysis) computational predictors can infer human traits with greater accuracy than we observed here. For example, the quantitative traits examined in our study were not adjusted for the known dependencies of traits on other variables (e.g., we might have corrected LDL cholesterol levels by age and sex [25]). Using trait values that have been adjusted for additional dependencies might better isolate those aspects of a trait that are attributable to patient genotype and therefore show improved correlation with predicted scores. However, restricting to the genetically determined component of traits seems unlikely a priori to favour one predictor over another, so we argue that the simpler analysis described here meets the goal of assessing predictors relative to one another.

Importantly, the *All of Us* dataset allowed us to benchmark performance in a racially and ethnically diverse population. While the UK Biobank cohort has strong demographic and ethnic biases (> 90% European ancestry) [50], more than 50% of participants in the *All of Us* cohort identify as racial and ethnic minorities. Assessing predictor performance in a diverse population is of critical importance if variant effect predictors are to be used to guide fundamental research (e.g., by empowering burden scanning) and as evidence in clinical variant classification [41]. That AlphaMissense was the top-performing predictor in both cohorts and that overall predictor rankings were similar will facilitate predictor choice in applications involving diverse populations. Future benchmarking efforts should continue to be extended in increasingly diverse population-level cohorts as they become available.

While the methodology we describe here was used to compare the performance of computational predictors to one another, we note that this approach could also be used to evaluate the growing body of experimental assay data used to infer variant effects (e.g., variant effect maps) [13, 51, 57], both to benchmark experimental evidence from multiple sources and to compare the relative performance of experimental and computational methods for inferring human traits. Finally, we note that each predictor was evaluated based only on its variant rankings, and that we did not evaluate the choice of score thresholds (e.g., from AlphaMissense or any other predictor) for the purpose of clinical variant classification.

## Conclusion

Computational variant effect predictors offer an increasingly reliable means of interpreting human genetic variation, but previous methods to evaluate and compare performance have been limited by concerns of circularity and bias. Our method to assess predictor performance, based on population-level cohorts of genotyped and phenotyped participants, sidesteps previous limitations. We applied this method to benchmark 24 computational variant effect predictors based on their ability to infer human traits in the UK Biobank and *All of Us* cohorts, finding AlphaMissense to be the top performer in each. The approach we outline here is generalizable to future predictors and can, therefore, continue to inform predictor choice for personal and clinical genetics.

## Methods

### Sequenced cohorts

This study was conducted with whole-exome sequencing data from the UK Biobank exome release (469,779 participants, UK Biobank field ID 23157) [4]. Variants were retrieved from the OQFE version [27, 40, 50] of the whole-exome VCF files (field ID 23157). The transfer of human data was approved and overseen by the UK Biobank Ethics Advisory Committee (project ID 51135). Participants who withdrew from the UK Biobank study (as of April 25th, 2023) were excluded from our sequenced cohort. For the remaining participants, the canonical isoform of each gene examined was defined according to the Ensembl database (GRCh38) [18], with exonic coding regions defined according to the CCDS database [37]. The corresponding coding variants were extracted from raw VCF files, with filtering adapted from the UK Biobank [50]: Phred quality score > 20, individual missingness < 10%, minimum read coverage depth of 7, and carried by at least one participant passed the allele balance threshold of 0.15. Variants were mapped to canonical transcripts and this set was further restricted to rare variants (MAF < 0.1%) in the gnomAD [23] and the UK Biobank cohorts.

Our orthogonal validation was conducted with short-read whole-genome sequencing data from the *All of Us* Controlled Tier Dataset v7 (245,400 participants). Post-sequencing, variant and sample QC was performed by the *All of Us* Data and Research Center [2]. The canonical isoform of each gene examined was defined according to the Ensembl database (GRCh38) [18]. The corresponding coding variants were extracted from Hail MatrixTables (version 0.2.107, Hail Team) and filtered for Phred quality score > 20, individual missingness < 10%, minimum read coverage depth of 7, and presence in at least one participant passed the allele balance threshold of 0.20. This set was further restricted to variants that were rare (MAF < 0.1%) in both the gnomAD v2 [23] and the *All of Us* cohorts.

### Phenotype processing

Phenotypes were extracted for all UK Biobank participants based on field IDs (listed in Additional file 1: Table S1). For a given phenotype, where participants had multiple measurements from repeat assessments, only measurements from the initial assessment were retained. All categorical phenotypes were treated as binary. Phenotypes for which less than 10 participants had a given trait, or for which there were less than 10 measurements for a given trait, in the case of quantitative phenotypes, were excluded from further analysis.

Phenotypes in the *All of Us* cohort were selected to match those from the UK Biobank set. Of the 55 UK Biobank traits, 43 had an equivalent measurement or set of measurements in *All of Us* (Additional file 1: Table S1) which resulted in 127 matching gene-trait combinations. Of these, a further 11 gene-trait combinations failed to pass the minimum participant cutoff of 10, and so were excluded. In all, 116 gene-trait combinations were matched between the UK Biobank and *All of Us* sets, comprising 87 genes and 38 traits. For each quantitative trait, units were harmonized and non-physiologic values were removed.

### Variant effect predictors

We considered 24 computational variant effect predictors (Additional file 2: Table S2). Precomputed scores were available for most predictors, many of which were retrieved from dbNSFP v4 (accessed May 2023) [29]. Pre-computed ESM1v scores, calculated as in [32], were kindly provided by B. Livesey and J. Marsh. Precomputed scores for CADD v1.7 [44] and MutPred2 [35] were kindly provided by T. Maass and M. Kircher and by V. Pejaver and his group, respectively. A predictor was only included in a comparison if it provided scores for at least 10 missense variants for a given gene. For predictors that assign low scores to predicted damaging variants (i.e., ESM1b, ESM-1v, FATHMM, LRT, PROVEAN and SIFT) scores were negated. All 24 predictors provided scores for more than 90% of the missense variants in both the UK Biobank and *All of Us* cohorts. All 24 predictors provided 10 or more predictions for 84/99 and 74/87 genes in the UK Biobank and *All of Us* sets, respectively.

### Predictor benchmarking

Predictor comparisons were conducted separately for each gene-trait combination, using different methods depending on whether the trait was binary or quantitative. For binary traits, predictor scores were rescaled to reduce the impact of outliers: we set a floor and ceiling at the 5th and 95th percentiles and normalized scores (0–1), with 0 corresponding to neutral variants and 1 corresponding to functionally damaging variants. We then computed a participant-centric variant score under an additive model (i.e., two missense variants with a score of 0.5 aggregate to a participant-centric score of 1). Predictor performance was assessed by measuring the area under the balanced precision-recall curve (AUBPRC). At a given score threshold $(s)$ precision is defined as $\frac{TP}{PP}$; where true positives $(TP)$ are participants with a variant score ≥ $s$ and a given trait, and predicted positives $(PP)$ are participants with variant scores ≥ $s$. Correspondingly, recall is defined as $\frac{TP}{P}$; where positives $(P)$ are participants with a given trait. Because precision is affected by the prevalence of positive events, we evaluated the balanced precision (i.e., the precision expected in a test set with an equal number of positive and negative entries) as described in Wu et al. [58]. The AUBPRC was calculated using the *yogiroc* R package [56]. For quantitative traits, where multiple participants harboured the same variant, trait values were averaged across all carriers and the pearson correlation coefficient (PCC) was measured. Here, each variant has one averaged trait value and one predicted variant effect score for a given predictor.

To estimate uncertainty in each of these measures, we carried out a 10 k-iteration bootstrap resampling (random sampling of participants with replacement) which generated a distribution of AUBPRC or PCC values for each predictor and each gene-trait combination. For quantitative traits, participants were resampled and variant-level mean trait values were recalculated for each sample. From this, we empirically determined the mean AUBPRC or PCC and the 95% CI of the distribution. To give equal weight to positive and negative correlations of similar strength, we used PCC^2^ instead of PCC.

For each of the 140 gene-trait combinations, we carried out a pairwise comparison of variant effect predictors based on mean performance values and calculated an empirical *p*-value for each pair. Here, for each predictor pair (e.g., predictors *a* and *b*), our empirical *p*-value is the fraction of measurements from the above resamplings where predictor *a* is outperformed by predictor *b* (i.e., $p\,value =\frac{\sum ((predictor a - predictor b) \le 0)}{i resamplings}$). To account for multiple hypothesis testing, we extracted the distribution of *p*-values (one for every pairwise comparison) and calculated Storey’s *q*-values to estimate the false discovery rate [48]. For each gene-trait combination, we consider the top-performing predictor to be the one with the highest mean performance and subsequently set an FDR threshold of 10%, above which a predictor is considered tied for best.

As an overall qualitative evaluation of each predictor, we summed the number of gene-trait combinations for which a predictor was either best performing (mean AUBPRC or PCC), or statistically indistinguishable from the numerically best predictor (FDR ≥ 10%). To assess quantitative performance differences between each pair of predictors, we performed a two-tailed Wilcoxon signed-rank test comparing mean performance measures across all gene-trait combinations. From this test statistic, we extracted *p*-values and calculated Storey’s *q*-values to correct for the false discovery rate. In this overall ranking, we deemed a predictor to have significantly outperformed another if the comparison yielded an FDR of less than 10%.

### Supplementary Information

Additional file 1. A list of the gene-trait combinations used to assess predictor performance in the UK Biobank and *All of Us* cohorts (Table S1).

Additional file 2. A list of the 24 predictors assessed in this study (Table S2).

Additional file 3. The number of variants (by gene) for which each predictor provided scores in the UK Biobank (Table S3) and All of Us (Table S4) cohorts.

Additional file 4. The genes for which predictors provided too few predictions.

Additional file 5. Review history.

## Data Availability

The source code used in our variant effect predictor benchmarking, as well as that used to extract and process participant data from the UK Biobank and *All of Us* cohorts, is available on GitHub (https://github.com/DanielTabet/VEP_benchmarking) under an MIT license [53], and an archived version has been made available on Zenodo [52]. The UK Biobank dataset is available by application via https://www.ukbiobank.ac.uk/. The *All of Us* Research Program’s Controlled Tier Dataset v7 is available to authorized users on the Researcher Workbench via https://www.workbench.researchallofus.org.

## References

[1] Adzhubei IA (2010). A method and server for predicting damaging missense mutations. Nat Methods.

[2] All of Us Research Program Investigators (2019). The ‘All of Us’ research program. New England J Med..

[3] Brandes N (2023). Genome-wide prediction of disease variant effects with a deep protein language model. Nat Genet.

[4] Bycroft C (2018). The UK Biobank resource with deep phenotyping and genomic data. Nature.

[5] Cheng J (2023). Accurate proteome-wide missense variant effect prediction with AlphaMissense. Science.

[6] Choi Y (2012). Predicting the functional effect of amino acid substitutions and indels. PLoS ONE.

[7] Chun S, Fay JC (2009). Identification of deleterious mutations within three human genomes. Genome Res.

[8] Cirulli ET (2020). Genome-wide rare variant analysis for thousands of phenotypes in over 70,000 exomes from two cohorts. Nat Commun.

[9] Cirulli ET, Goldstein DB (2010). Uncovering the roles of rare variants in common disease through whole-genome sequencing. Nat Rev Genet.

[10] Curtis D (2020). Multiple linear regression allows weighted burden analysis of rare coding variants in an ethnically heterogeneous population. Hum Hered.

[11] Kuang Da (2021). MaveRegistry: a collaboration platform for multiplexed assays of variant effect. Bioinformatics.

[12] Dong C (2015). Comparison and integration of deleteriousness prediction methods for nonsynonymous SNVs in whole exome sequencing studies. Hum Mol Genet.

[13] Fowler DM (2023). An Atlas of Variant Effects to understand the genome at nucleotide resolution. Genome Biol.

[14] Frazer J (2022). Publisher Correction: Disease variant prediction with deep generative models of evolutionary data. Nature.

[15] Garber M (2009). Identifying novel constrained elements by exploiting biased substitution patterns. Bioinformatics.

[16] Grimm DG (2015). The evaluation of tools used to predict the impact of missense variants is hindered by two types of circularity. Hum Mutat.

[17] Hobbs HH, Brown MS, Goldstein JL (1992). Molecular genetics of the LDL receptor gene in familial hypercholesterolemia. Hum Mutat.

[18] Howe KL (2021). Ensembl 2021. Nucleic Acids Res.

[19] Ioannidis NM (2016). REVEL: An Ensemble Method for Predicting the Pathogenicity of Rare Missense Variants. Am J Hum Genet.

[20] Ionita-Laza I (2016). A spectral approach integrating functional genomic annotations for coding and noncoding variants. Nat Genet.

[21] Jagadeesh KA (2016). M-CAP eliminates a majority of variants of uncertain significance in clinical exomes at high sensitivity. Nat Genet.

[22] Jurgens SJ (2022). Analysis of rare genetic variation underlying cardiometabolic diseases and traits among 200,000 individuals in the UK Biobank. Nat Genet.

[23] Karczewski KJ (2020). The mutational constraint spectrum quantified from variation in 141,456 humans. Nature.

[24] Katsonis P (2022). Genome interpretation using in silico predictors of variant impact. Hum Genet.

[25] Khera AV (2016). Diagnostic Yield and Clinical Utility of Sequencing Familial Hypercholesterolemia Genes in Patients With Severe Hypercholesterolemia. J Am Coll Cardiol.

[26] Kircher M (2014). A general framework for estimating the relative pathogenicity of human genetic variants. Nat Genet.

[27] Krasheninina, O. *et al.* (2020) “Open-source mapping and variant calling for large-scale NGS data from original base-quality scores,” *bioRxiv*. 10.1101/2020.12.15.356360.

[28] Lee S (2014). Rare-variant association analysis: study designs and statistical tests. Am J Hum Genet.

[29] Liu X (2020). dbNSFP v4: a comprehensive database of transcript-specific functional predictions and annotations for human nonsynonymous and splice-site SNVs. Genome medicine.

[30] Livesey BJ, Marsh JA (2020). Using deep mutational scanning to benchmark variant effect predictors and identify disease mutations. Mol Syst Biol.

[31] Livesey BJ, Marsh JA. Interpreting protein variant effects with computational predictors and deep mutational scanning. Dis Models Mech 2022;15(6). 10.1242/dmm.049510. 10.1242/dmm.049510 PMC9235876 35736673

[32] Livesey BJ, Marsh JA (2023). Updated benchmarking of variant effect predictors using deep mutational scanning. Mol Syst Biol.

[33] Lu Q (2015). A statistical framework to predict functional non-coding regions in the human genome through integrated analysis of annotation data. Sci Rep.

[34] Meier J. et al. Language models enable zero-shot prediction of the effects of mutations on protein function. 2021. “*bioRxiv*. 10.1101/2021.07.09.450648.

[35] Pejaver V (2020). Inferring the molecular and phenotypic impact of amino acid variants with MutPred2. Nat Commun.

[36] Povysil G (2019). Rare-variant collapsing analyses for complex traits: guidelines and applications. Nat Rev Genet.

[37] Pujar S (2018). Consensus coding sequence (CCDS) database: a standardized set of human and mouse protein-coding regions supported by expert curation. Nucleic Acids Res.

[38] Qi H (2021). MVP predicts the pathogenicity of missense variants by deep learning. Nat Commun.

[39] Quang D, Chen Y, Xie X (2015). DANN: a deep learning approach for annotating the pathogenicity of genetic variants. Bioinformatics.

[40] Regier AA (2018). Functional equivalence of genome sequencing analysis pipelines enables harmonized variant calling across human genetics projects. Nat Commun.

[41] Richards S (2015). “Standards and guidelines for the interpretation of sequence variants: a joint consensus recommendation of the American College of Medical Genetics and Genomics and the Association for Molecular Pathology”, *Genetics in medicine: official journal of the American College of Medical Genetics*. Nat Publ Group.

[42] Riesselman AJ, Ingraham JB, Marks DS (2018). Deep generative models of genetic variation capture the effects of mutations. Nat Methods.

[43] Samocha KE. et al. Regional missense constraint improves variant deleteriousness prediction. 2017. *bioRxiv*. 10.1101/148353.

[44] Schubach M (2024). CADD v1.7: using protein language models, regulatory CNNs and other nucleotide-level scores to improve genome-wide variant predictions. Nucleic Acids Res.

[45] Schwarz JM (2014). MutationTaster2: mutation prediction for the deep-sequencing age. Nat Methods.

[46] Shihab HA (2013). Predicting the functional, molecular, and phenotypic consequences of amino acid substitutions using hidden Markov models. Hum Mutat.

[47] Starita LM (2017). Variant Interpretation: Functional Assays to the Rescue. Am J Hum Genet.

[48] Storey JD (2002). A direct approach to false discovery rates. J Royal Stat Soc Ser B, Stat Methodol.

[49] Sundaram L (2018). Predicting the clinical impact of human mutation with deep neural networks. Nat Genet.

[50] Szustakowski JD (2021). Advancing human genetics research and drug discovery through exome sequencing of the UK Biobank. Nat Genet.

[51] Tabet D (2022). Scalable functional assays for the interpretation of human genetic variation. Annu Rev Genet.

[52] Tabet D, Kuang D. DanielTabet/VEP_benchmarking: VEP benchmarking. 2024. Zenodo. 10.5281/zenodo.11359196.

[53] Tabet D, Kuang D. *VEP_benchmarking: Benchmarking variant effect predictors in population-based cohorts*. Github. 2024b. Available at: https://github.com/DanielTabet/VEP_benchmarking.

[54] Van Hout CV (2020). Exome sequencing and characterization of 49,960 individuals in the UK Biobank. Nature.

[55] Vaser R (2016). SIFT missense predictions for genomes. Nat Protoc.

[56] Weile, J. (2021) *yogiroc: Simple ROC and PRC curves*. Available at: https://github.com/jweile/yogiroc (Accessed: 11 Mar 2022).

[57] Weile J, Roth FP (2018). Multiplexed assays of variant effects contribute to a growing genotype–phenotype atlas. Hum Genet.

[58] Wu Y (2021). Improved pathogenicity prediction for rare human missense variants. Am J Hum Genet.

