# Supplementary material for: Benchmarking computational variant effect predictors by their ability to infer human traits
Source: Genome Biol. 2024 Jul 1;25:172. doi: 10.1186/s13059-024-03314-7 (PMC11218265; doi:10.1186/s13059-024-03314-7)
Supplement: Supplementary file 5 — Additional file 5. Review history. [file 13059_2024_3314_MOESM5_ESM.docx]

Review History

**First round of review**

**Reviewer 1**

**Were you able to assess all statistics in the manuscript, including the appropriateness of statistical tests used?**

No, I do not feel adequately qualified to assess the statistics.

**Comments to author:**

This paper performed a comprehensive evaluation of 20 variant effect predictors on the UK Biobank data, which kick a key issue on rare variant detection and related function prediction.
However, the limitation of this study is not include all published predictors, but without fully compared these 20 method's pros and cons, and didn't considered how sample size influence the prediction results. And my option is major reversion with following comments:

Majors:
1. All 20 variant effect predictors should be clearly described and catered to several types, which other people can understand these methods quickly, and comparison results would show which type method could enhance the power of rare variant detection and function prediction.
2. If the author can add more dataset, not only the UK biobank data, the results would be more fair.
3. These methods would be sensitive to sample size, and a simulation results of these 20 methods works on random select 10K, 50k, 100k, 200K, 450K from the selected 450k UK biobank dataset would help to show this ability.
4. Organization of the main text is not clear, need a well organized with logically way writing.
5. For a methodology paper, an open source codes at GitHub or somewhere for repeatable is supper important.

Miniors:
1. The formation of citations were in a chaotic way.

**Reviewer 2**

**Were you able to assess all statistics in the manuscript, including the appropriateness of statistical tests used?**

No, I do not feel adequately qualified to assess the statistics.

**Comments to author:**

The authors have investigated performance of variant effect (pathogenicity/tolerance) predictors with sequence and trait information from UK Biobank with traits to predict burden of variants. They tested 140 gene-trait combinations and extended the two best performing methods to analysis of about 450 000 biobank individuals. 20 variant interpretation algorithms were tested. The obtained results were correlated to information for cardiovascular diseases.

Burden tests are quite widely used and test with non-circulatory data set provides insight on the performance of the tools. The used data set is unique in studies in this field.

The approach is based on the assumption that several rare variants in a trait related gene would be predicted to be harmful/disease-elated. This is likely true, however, the vulnerability of genes for variations varies widely and there are numerous genes, which although disease-related, do not contain many harmful variants. There may be a handicap for methods that do not predict (close to) all variants.

The used data is quite small in size. It represents the currently available data, but some differences might be noted with larger set, once available.
Several of the traits are complex, thereby the experimental data

Page 12. Description of protein-truncating variants would be needed. Due to NMD such variants are often not expressed at all and it would be wrong to call missing proteins as truncated ones.

The used model and design principle assumes variants to be either pathogenic or benign, however, this is not true as the phenotype can depend on many other factors and a certain genotype in different individuals can cause different phenotypes.

The used data is sensitive, and thus not publicly available. However, it would be very difficult to repeat or use the data (with permission) for further studies since the users would need to repeat the selections that authors made. There are hardly enough details to facilitate that, and it would be unnecessary. The authors should consider to store the made selections and share them with the permission of the Biobank or even store them into the biobank.

**Authors Response**

**Point-by-point responses to the reviewers’ comments:**

We are grateful for your thoughtful and constructive comments. Below we list editorial and reviewer comments in *italics* with our responses in blue text, addressing each of the issues raised and describing the corresponding changes made to the revised manuscript.

Broadly, we present a restructured manuscript in which we 1) update our predictor benchmarking to include recently published methods (including AlphaMissense, ESM1b, and ESM1-v); 2) replicate this benchmarking in the independent whole-genome sequenced *All of Us* cohort; 3) remove burden scan results from the current manuscript pending validation in an orthogonal cohort.

*Reviewer #1: This paper performed a comprehensive evaluation of 20 variant effect predictors on the UK Biobank data, which kick a key issue on rare variant detection and related function prediction.*

*However, the limitation of this study is not include all published predictors, but without fully compared these 20 method's pros and cons, and didn't considered how sample size influence the prediction results. And my option is major reversion with following comments:*

*Majors:*

1. *All 20 variant effect predictors should be clearly described and catered to several types, which other people can understand these methods quickly, and comparison results would show which type method could enhance the power of rare variant detection and function prediction.*

We thank the reviewer for their comment. The revised manuscript now cites high-quality reviews which provide in-depth overviews of computational variant effect predictor methods. At the Editor’s discretion, we would be happy to expand the introduction, but given that this would greatly lengthen the manuscript while duplicating the content of previous reviews, we have not yet done this. At the reviewer’s suggestion, the revised manuscript discusses trends in which types of predictors fared best in the comparison.

2. *If the author can add more dataset, not only the UK biobank data, the results would be more fair.*

We agree with the reviewer that validation in an independent cohort is of great importance. We have therefore also carried out our computational predictor comparison in the *All of Us* cohort, an independent whole-genome sequenced and phenotyped cohort of ~250k participants of diverse racial and ethnic backgrounds.

3. *These methods would be sensitive to sample size, and a simulation results of these 20 methods works on random select 10K, 50k, 100k, 200K, 450K from the selected 450k UK biobank dataset would help to show this ability.*

We thank the reviewer for their comment. Although we have removed the burden scan aspects of our study, largely obviating this concern, we note that the revised manuscript now repeats predictor benchmarking with the independent *All of Us* cohort, which is approximately half the size of the UK Biobank dataset (245k vs 465k participants).

4. *Organization of the main text is not clear, need a well organized with logically way writing.*

We regret that the reviewer found the study difficult to follow and have simplified and improved the overall logical flow of the manuscript.

5. *For a methodology paper, an open source codes at GitHub or somewhere for repeatable is supper important.*

We completely agree, and have made all code available on GitHub at [https://github.com/DanielTabet/VEP_benchmarking.](https://github.com/DanielTabet/VEP_benchmarking)

*Minors:*

*1.	The formation of citations were in a chaotic way.*

We have reviewed all citation formatting in the revised manuscript, and trust that any remaining errors will be caught by the production editorial staff.

———

Reviewer #2: *The authors have investigated performance of variant effect (pathogenicity/tolerance) predictors with sequence and trait information from UK Biobank with traits to predict burden of variants. They tested 140 gene-trait combinations and extended the two best performing methods to analysis of about 450 000 biobank individuals. 20 variant interpretation algorithms were tested. The obtained results were correlated to information for cardiovascular diseases.*

*Burden tests are quite widely used and test with non-circulatory data set provides insight on the performance of the tools. The used data set is unique in studies in this field.*

*The approach is based on the assumption that several rare variants in a trait related gene would be predicted to be harmful/disease-elated. This is likely true, however, the vulnerability of genes for variations varies widely and there are numerous genes, which although disease-related, do not contain many harmful variants. There may be a handicap for methods that do not predict (close to) all variants.*

We thank the reviewer for their comment. The revision now notes that each of the 24 computational predictors in our assessment provided scores for > 90% of the variants in our set. Our study also eliminated one method (EVE) from our comparison because of precisely this concern.

*The used data is quite small in size. It represents the currently available data, but some differences might be noted with larger set, once available.*

This point is especially critical for the statistical power of the burden scan we previously described, but this is no longer relevant given that those results were removed from the current manuscript so as to focus more clearly on the predictor comparison. While a larger cohort could increase the statistical power of our predictor benchmarking, we believe the size of the UK Biobank cohort is sufficient to assess the performance of computational predictors relative to one another. Indeed, as the reviewer notes, this is the largest currently available well-phenotyped exome-sequenced cohort, and we now find consistent results in an independent cohort.

*Several of the traits are complex, thereby the experimental data*

It appears that the end of this comment is missing, we await guidance from the editor.

*Page 12. Description of protein-truncating variants would be needed. Due to NMD such variants are often not expressed at all and it would be wrong to call missing proteins as truncated ones.*

We thank the reviewer for their comment and note that, since the burden scan results have been removed from the current manuscript, this comment is no longer applicable.

*The used model and design principle assumes variants to be either pathogenic or benign, however, this is not true as the phenotype can depend on many other factors and a certain genotype in different individuals can cause different phenotypes.*

As the burden scanning aspect of the manuscript has been removed from the revision, this concern is no longer applicable.

*The used data is sensitive, and thus not publicly available. However, it would be very difficult to repeat or use the data (with permission) for further studies since the users would need to repeat the selections that authors made. There are hardly enough details to facilitate that, and it would be unnecessary. The authors should consider to store the made selections and share them with the permission of the Biobank or even store them into the biobank.*

We agree that reproducibility is challenging where private datasets are concerned. In the absence of a dedicated system to share sensitive datasets, we have noted in the manuscript that all code used to extract and process patient data from the UK Biobank and *All of Us* cohorts are now available via [https://github.com/DanielTabet/VEP_benchmarking.](https://github.com/DanielTabet/VEP_benchmarking)

**Second round of review**

**Reviewer 1**

I happy with the author's update with extended dataset and methods, especially include AlphaMissense into the comparison, which updated all my concerns. I suggest the manuscript is acceptable after strict format check.
